# New Gene Therapy Strategy for β-Thalassemia

**DOI:** 10.1007/s12015-026-11132-6

**Published:** 2026-04-23

**Authors:** Dongguo Liang, Ingo G.H. Schmidt-Wolf, Jingjing Pu

**Affiliations:** 1 Shanghai Institute of Hematology, National Research Center for Translational Medicine, State Key Laboratory of Medical Genomics, Ruijin Hospital, Shanghai Jiao Tong University School of Medicine, Shanghai, 20025 China; 2 Department of Hematology, Renji Hospital, Shanghai Jiao Tong University School of Medicine, Shanghai, 200127 China; 3 Department of Integrated Oncology, Center for Integrated Oncology (CIO) Bonn, University Hospital Bonn, NRW, Bonn, 53127 Germany

**Keywords:** β-thalassemia, Hemoglobin switching, Fetal hemoglobin (HbF) reactivation, Genome editing, *BCL11A*

## Abstract

β-thalassemia is a common inherited hemoglobin disorder caused by reduced or absent β-globin production, leading to ineffective erythropoiesis, chronic anemia, and, in severe cases, lifelong transfusion dependence. Although allogeneic hematopoietic stem cell transplantation can be curative, its use is limited by donor availability and transplant-related complications. In recent years, gene therapy has emerged as a promising alternative and has rapidly changed the treatment landscape for β-thalassemia. In this review, we summarize both established and emerging gene-based strategies, including lentiviral gene addition to restore *HBB* expression and gene editing approaches aimed at reactivating fetal hemoglobin. We discuss key targets such as the erythroid-specific *BCL11A* enhancer, repressor-binding sites in the *HBG* promoters, and other regulatory elements involved in globin switching. We also highlight the growing potential of newer technologies such as base editing and prime editing, which may offer greater precision and reduce the risks associated with double-strand DNA breaks. Finally, we address the major challenges that still need to be resolved, including safety, durability, technical complexity, and access to treatment. Overall, gene therapy is moving β-thalassemia closer to a broadly applicable curative approach.

## Introduction

Hemoglobin, a heterotetramer containing two α-and two β-globin subunits, transports oxygen in our body. The β-like globin subunits are encoded by the five functional genes in the β-globin locus. During human development, these β-like globin genes are sequentially activated (from ε (embryonic) to γ (fetal) and then to β (adult)), and the composition of hemoglobin coincidentally changes [1]. Sequential globin regulation is called the hemoglobin switching: the embryonic globin gene is expressed first in the yolk sac, and then the fetal globin gene is activated in the spleen and liver while the embryonic gene is gradually silenced. Finally, expressed genes are switched from the fetal globin genes to the adult type mainly produced in the bone marrow [2]. Quantitative mutations in the adult type of globin genes result in thalassemia, and qualitative counterparts cause sickle cell disease (SCD). According to the affected globin subunits and underlying genetic mutations, thalassemia can be further classified into α-thalassemia and β-thalassemia. β-thalassemia is characterized by reduced or no production of the β-globin chain, a condition which causes the accumulation of excess, unstable α-globin chains that precipitate as inclusion bodies [1], leading to ineffective erythropoiesis and premature hemolysis of circulating red cells. Further, β-thalassemia is one of the most pervasive monogenic disorders around the world. β-thalassemia carriers as estimated to account for approximately 1.5% of the population around the world, and every year approximately 40,000 newborns are diagnosed, half of whom are transfusion dependent [3]. Most β-thalassemia patients are distributed along the equator, which encompasses mainly low-income countries; however, immigration and other factors have gradually increased the incidence of β-thalassemia in Europe and North America [4, 5. Given their severity and prevalence, monogenic disorders impose a heavy healthcare burden on our society, particularly in low- or middle-income countries, most of which have next to no facilities for diagnosing, controlling, and managing the common hemoglobin disorders [6, 7]. To optimize its management, β-thalassemia is now classified into transfusion-dependent β-thalassemia (TDT) and non–transfusion-dependent β-thalassemia (NTDT). Conventional treatment of β-thalassemia includes blood transfusion as well as iron chelation, and allogeneic hematopoietic stem cell transplantation (allo-HSCT) is currently the only radical method [8], but the difficulty of donor matching and high cost limit the application of allo-HSCT. In recent years, with the continuous deepening of research on the pathophysiological mechanism of β-thalassemia, especially the greater understanding of the regulatory mechanism of the hemoglobin switching in humans, the management of β-thalassemia has ushered in new era, such as gene therapy that targets the diverse transcriptional suppressors of the γ-globin gene. This review mainly focuses on summarizing and discussing the advances in research on the hemoglobin switching and novel therapeutic approaches targeting these targets.

## Hemoglobin switching and Molecular Regulation

**Fig. 1:**
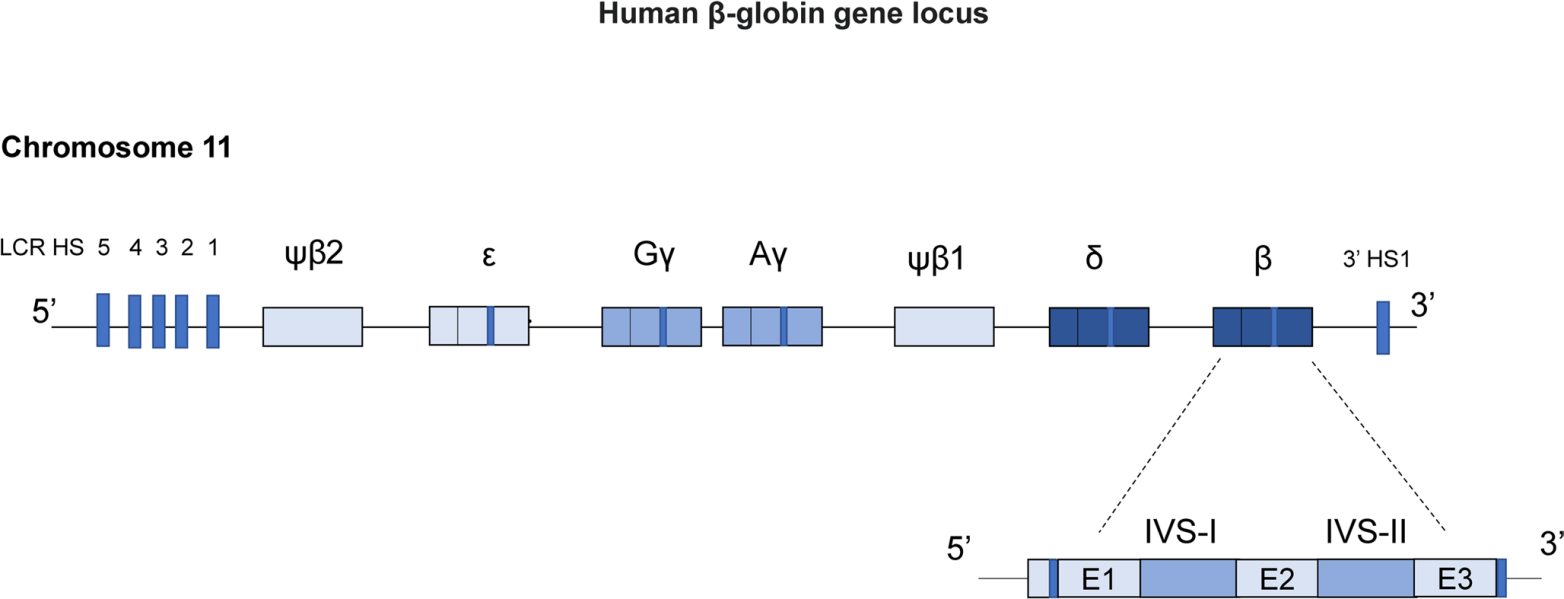
The light blue box indicates the gene (ε-globin) expressed during the embryonic stage; the medium blue boxes represent the genes (Gγ-globin, and Aγ-globin) activated during the fetal stage; and the deep blue boxes are the genes (δ-globin and β-globin) expressed during adulthood. IVS=intervening segments (or introns). E1, E2 and E3 denote the three exons

**Fig. 2:**
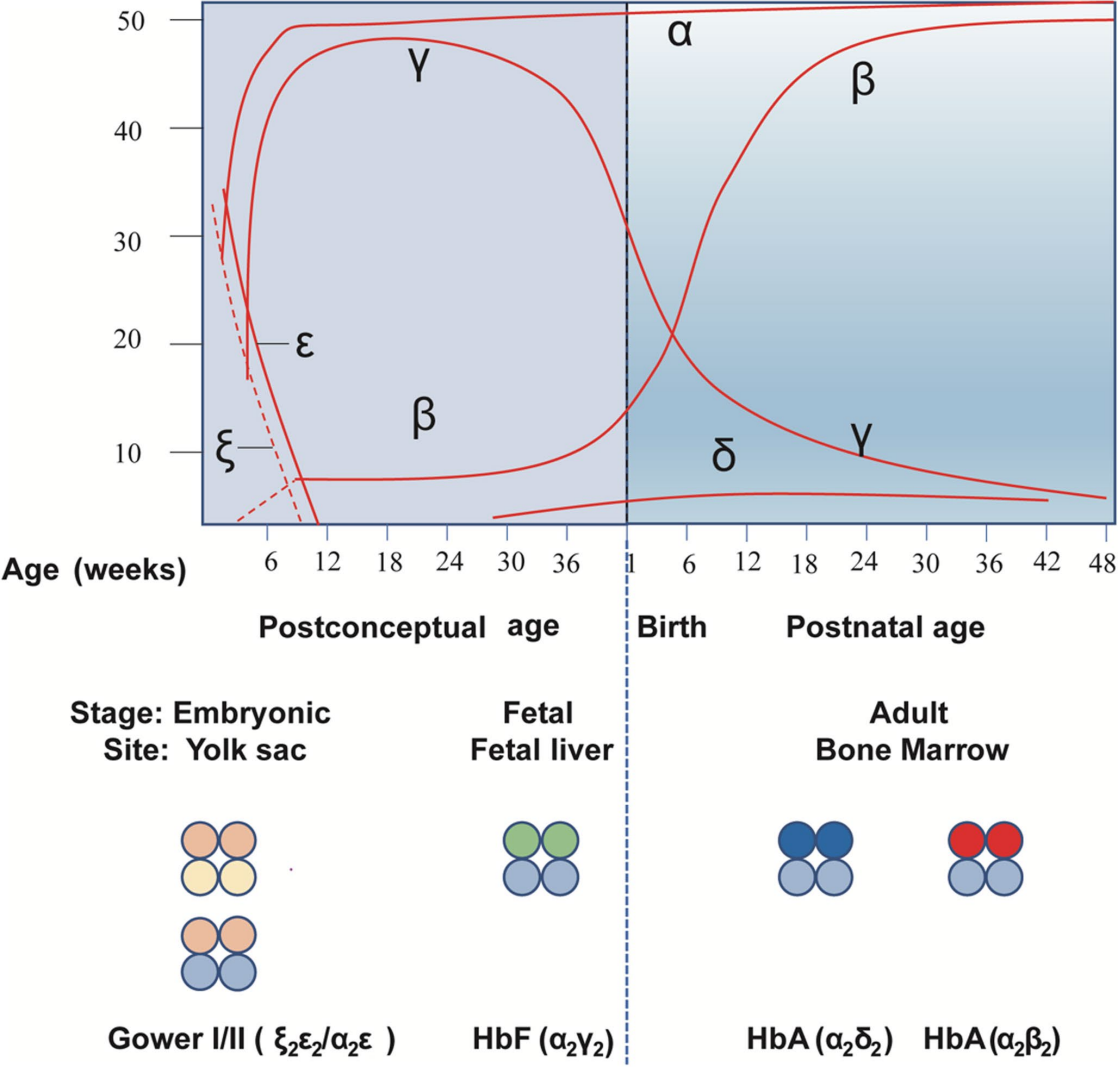
Hemoglobin switching during human development. Hemoglobin switching parallels erythropoietic development, progressing from embryonic forms (yolk sac) to fetal hemoglobin (HbF, liver), and finally to adult hemoglobin (HbA) after birth, with the γ- to β-globin transition initiating around mid-gestation and completing in early infancy

The human β-globin gene locus is situated on chromosome 11p15.5 [9], containing five functional genes (*HBE*, *HBG2*, *HBG1*, *HBD*, and *HBB*) and one pseudogene (*HBBP1*) that are arranged according to their developmental expression in red cells [10]. Each β-globin gene has two introns and three coding exons, with a total of 146 amino acids [11]. Upstream of the gene cluster stands a locus control region (LCR), consisting of five DNase I hypersensitive sites (HSs) (5′HS1–5′HS5), which may play a vital role in the hemoglobin switching [12, 13]. In addition, downstream of the β-globin gene locus exists a single hypersensitive site, 3’HS1. As a key CTCF-dependent insulator and chromatin architectural element, 3’HS1 participates in forming the active chromatin hub by looping with the locus control region (LCR) and globin gene promoters, and critically modulates the developmental silencing of γ-globin and expression balance of the β-globin cluster [14–16](Fig. 1). Gene expression and switching on the human β-globin gene locus are parallel to the developmental transition of erythropoiesis sites. At the beginning, embryonic hemoglobins (ζ_2_ε_2_, α_2_ε_2_, and ζ_2_γ_2_) first emerge in the yolk sac and dominate in red cells during early gestation. Then, the fetal hemoglobin (HbF [α_2_γ_2_]) gradually becomes the major component of erythroid cells, during which the erythropoiesis site switches from the yolk sac to the spleen and liver. The most key transition from γ-globin gene to β-globin gene starts at approximately the 12th week of gestation and is accomplished at 6 months old, after which most of the hemoglobin (> 95%) in erythroid cells consists of adult hemoglobin (HbA [α_2_β_2_]), with minor concentrations of HbA2 (α_2_δ_2_) (2.0–3.5%) and HbF (< 1.0%)^8^(Fig. 2). Notably, the symptoms of β-thalassemia do not appear until the silencing of the fetal hemoglobin gene around birth, although the adult β-globin gene cannot express normally from the very beginning [17]. Furthermore, the hereditary persistence of fetal hemoglobin (HPFH) can relieve anemia severity when co-inherited with β-thalassemia, as increased HbF production (α₂γ₂) compensates for the reduced β-globin by pairing with excess α-globin chains and reducing globin chain imbalance [17, 18]. All these phenomena suggest that reactivation of γ-globin gene expression is a very promising direction to treat β-thalassemia; consequently, the study of the regulatory mechanism of the hemoglobin switching has attracted geneticists and hematologists for decades. Given the in-depth research and new discoveries on the regulation mechanism of the hemoglobin switching in recent years, gene therapy targeting fetal globin gene transcriptional factors has achieved amazing results, which has revolutionized β-thalassemia treatment.

### LCR

The LCR, located upstream of the β-globin gene locus and consisting of five DNase I hypersensitive sites (HSs), was discovered in the early 1980 s and accepted as a term in 1990 at The 7th Conference on Hemoglobin Switching [19]. Each HS has a core sequence that distributes some motifs for transcriptional regulators; and the motifs for NFE2 and GATA-1 exist in all 5 HSs [20]. These HSs have special functions. For example, HS5 acts as an insulator, isolating the globin gene locus from the surrounding inactive chromatin [21]; further, HS3 can activate ε and γ-globin gene expression [22]. The most important aspect of the LCR is that it enhances the β like globin genes and endows them with stage-specific expression [20]. As to how LCR interacts with the downstream globin genes, several hypotheses attempt to explain this biological phenomenon, including looping [23], tracking [24], facilitated tracking [25], and linking [26] models. Among these schemes, the looping model proposes that the HSs of the β-globin LCR can fold to form a complex, with the HS core elements creating an active site that binds transcription factors (TFs). This theory greatly stimulates and inspires researchers to explore the interplay between genes and remote regulatory elements [20].

## Transcription factors/modulators and the hemoglobin switching

The hemoglobin switching is now held to be simultaneously regulated by multiple cis-acting elements and trans-acting factors, and three cis-acting elements are identified to be associated with this process: *BCL11A*, β-globin loci, and HBS1L-MYB [27]. Initially, knowledge of the mechanism of the hemoglobin switching was gained mainly from studying the natural regulatory mutations of *HPFH*. Through genome-wide association studies (GWAS) and subsequent functional research, a zinc-finger transcriptional factor, B-cell lymphoma/leukemia 11 A (*BCL11A*), was identified as a major suppressor of the fetal globin gene [27–29], which has been a main target of gene therapy for β-thalassemia. In 2016, another key suppressor, transcription factor Zinc Finger And BTB Domain Containing 7 A (*LRF*/*ZBTB7A*) was found to repress the expression of fetal hemoglobin independent of *BCL11A [*30], both of which can directly bind the γ-globin gene (*HBG*) promoter [31]. More recently, Krüppel-like factor 1 (*KLF1*/*EKLF*) was reported to reactivate *HBG* expression in British *HPFH*. The British *HPFH* mutation can create a *de novo* recognition site for *KLF1*, suggesting *KLF1* as a potent erythroid activator [32]. However, some reports have indicated that *KFL1* plays an inhibitory role in the expression of *HBG* and can promote the expression of β-globin gene (*HBB*) [33–38]. The same situation also occurs for *GATA1*. How should we interpret these conflicting findings? We continue to elaborate on and discuss these TFs below.

The silencing of the γ-globin genes is mediated by a combined action of many factors (Tables 1 and 2), among which *BCL11A* and the *LRF* are identified as the foremost erythroid-specific HbF repressors [28, 30] which can directly bind the fetal globin gene promoter [31, 39]. Epigenetic modulators such as the lysine-specific demethylase 1 and repressor element-1 silencing transcription factor corepressor 1 (LSD1/CoREST) histone demethylase complex, nucleosome remodeling and deacetylase (NuRD) complex, lysine demethylase 1 A (*KDM1A*), DNA methyltransferase 3 A (*DNMT3A*), and euchromatic histone lysine methyltransferase (*EHMT*), together with lineage-defining factors such as *GATA1*, *MYB*, and *KLF1*, have an important role in coordinating the function of *BCL11A* or the *LRF [*40–44]. Furthermore, genetic reactivation of *HBG* is mediated by *LIN28B* via disruption of the *BCL11A* mRNA directly^45^, suggesting epigenetic mechanisms or post-transcriptional regulation in the control of γ-globin gene expression.

**Table 1:** Transcription factors/modulators for regulating the hemoglobin switching

Transcription factors/Modulators	Mechanism
*BCL11A*	Directly binds to *HBG* promoter to promote interaction between LCR and *HBB*
*LRF/ZBTB7A*	Occupies the fetal globin gene promoter and maintains nucleosome density required for HBG silencing independent of BCL11A
*KLF1/EKLF*	Directly binds the β-globin and BCL11A promoters to activate both genes
*ZNF410/APA-1*	Directly targets CHD4, a component of the NuRD complex
*NFI*	Increases the expression of *BCL11A*, and directly suppresses the *HBG1*/*2* genes;
*HRI*	Erythroid-specific kinase activating *ATF4* to promote *BCL11A* transcription and fetal hemoglobin silencing;
*ETS2*	Directly binds UEBS and DEBS (upstream of *HBG2* and downstream of *HBG1*), mediating epigenetic regulation independent of known erythroid repressor complexes
*PTEN*	Suppresses HbF through the negative control of AKT signaling;
*HIF1α*	Activates via BGLT3 binding, recruiting transcriptional activators, promoting chromatin opening and enhancing γ-globin–LCR interactions
*BAP1*	DRED repressor subunit mediating site-specific *NCoR1* recruitment within the β-globin locus
*ETO2*	Represses the LDB1 complex and interacts with the NuRD complex, reshaping the transcriptional and epigenetic landscape to remodel chromatin organization
*NonO*	Directly interacts with SOX6 and binds directly to the ATGCAAAT core motif of the γ-globin proximal promoter
*HIC2*	Disrupts *GATA1* binding at the *BCL11A* enhancer, reducing promoter–enhancer interaction
NuRD Complex	Epigenetic repressor of fetal globin genes in concert with *BCL11A*

**Table 2:** Modulators involved in the hemoglobin switching in concert with *BCL11A* or the NuRD complex

Proteins interaction with BCL11A	NuRD complex	LDB1 complex
**TFs**: *GATA1* *SOX6* FOG-1(*ZFPM1*) *RUNX1* IKAROS (*IKZF1*) **NuRD complex** **LSD1/CoREST complex**: LSD1 (*KDM1A*) CoREST (*RCOR1*) **NCoR/SMRT complex**: NCoR (*NCOR1*) SMRT (*NCOR2*) *TBLR1* *TBL1* *CORO2A* KAISO (*ZBTB33*) **SIN3 complex**: *SIN3A* *SIN3B* **Other corepressors**: *BCOR* *TRIM28* **Other nuclear factors**: *DNMT1* JMJD1A (*KDM3A*) JMJD1B (*KDM3B*) JARID1A (*KDM5A*) *CARM1* *YLPM1* *MSH2* **SWI/SNF complex**: SNF5 (*SMARCB1*) BRG1 (*SMARCA4*) BRM (*SMARCA2*) BAF57 (*SMARCE1*) BAF60A (*SMARCD1*) BAF60B (*SMARCD2*) BAF155 (*SMARCC1*) BAF170 (*SMARCC2*) SNF2H (*SMARCA5*) BAF180 (*PB1*) *ASH2L* **Nuclear matrix**: MATRIN-3 (*MATR3*)	*HDAC1* *HDAC2* Mi-2α (*CHD3*) Mi2β (*CHD4*) *RBBP4* *RBBP7* *MTA1* *MTA2* *MTA3* *MBD2* *MBD3* P66α (*GATAD2A*) P66β (*GATAD2B*) --------------------------- **Proteins interaction with NuRD complex**: *TR2/TR4* *DNMT1* *ETO2* *GATA1*	*LDB1* *GATA1* *TAL1* *LMO2*

### BCL11A: major*HBG* suppressor

*BCL11A* was originally found to be a vital factor for the normal development of B lymphocytes [46] and also plays an important role in malignant tumors of the lymphatic system [47]. Later, GWAS analyses in individuals with HPFH identified BCL11A as a locus associated with the phenotype [27, 29]. *BCL11A* is expressed in the manner of developmental stages and is identified as a key regulatory suppressor of the *HBG* gene [28]. It binds to the distal TGACCA sequence of the *HBG* gene promoter (− 118 to − 113) with its three C-terminal zinc fingers (ZFs) [48]. Furthermore, *BCL11A*, together with its co-repressors, represses γ-globin expression by limiting interactions between the LCR and the *HBG* genes and favoring interaction between the LCR and *HBB*, thereby promoting hemoglobin switching [39]. The TGACCA sequence is present in the promoters of human and mouse embryonic as well as fetal globin genes. Notably, such a sequence is not found in the promoter of adult globin genes. In addition, two TGACCA sequences exist (−118 to −113, −91 to − 86) in each human *HBG* gene. In HPFH individuals, point mutations (−117, −114) or 13 bp deletion in the distal TGACCA sequence leads to the inability of *BCL11A* to bind to this sequence, resulting in the continuous expression of *HBG [*29, 49–51].

*BCL11A* belongs to the C2H2 zinc finger protein family, with multiple alternative splicing. In adult erythrocytes, *BCL11A-XL* and *BCL11A-L* are the main expression forms [28, 52. The inhibitory effect of *BCL11A-XL* on *HBG* expression was more significant than that of *BCL11A-L*. Compared with *BCL11A-L*, *BCL11A-XL* has an additional C-terminal ZF4-6 fragment. The ZF4-6 fragment of *BCL11A* has a much higher affinity to TGACCA than that of ZF23 to TNCGGCCA, thus determining the binding tendency sequence of the entire protein [39]. Although the ZF4-6 region of the BCL11A protein determines its DNA-binding preference, the N-terminal segment of *BCL11A* (amino acids 1–10) and ZF23 are also required for suppression of *HBG* expression, possibly through recruitment of additional TFs and RNA into a repressive complex [39]. The N-terminal segment of *BCL11A* contains the binding site of NuRD[53], and NuRD down-regulation can induce *HbF* expression [44]. Currently, the crystal structure of the BCL11A ZF4-6 domain in complex with its target DNA sequence in the *HBG* promoter has been elucidated, improving our understanding of the molecular mechanism by which BCL11A represses γ-globin expression [48].

The *BCL11A* recognition site TGACCA overlaps with the *HBG* activator NF-Y/alpha-CP1 binding site CCAAT sequence. During embryonic and fetal periods, NF-Y can bind to its target sequence, mediating the interplay between LCR and *HBG* promoter owing to the low expression of *BCL11A [*54, 55]. In adulthood, *BCL11A* is highly expressed and has a preferential binding to its recognition sequence than that of NF-Y to the CCAAT sequence. Given the steric hindrance effect, the combination of NF-Y and the *HBG* promoter is blocked, thereby promoting the interplay between LCR and the promoter of the *HBB* gene, leading to the fetal-to-adult hemoglobin switching [54, 55].

Multiple factors can regulate *BCL11A* expression, and, in turn, these agents control the developmental hemoglobin switching. HIC ZBTB Transcriptional Repressor 2 (*HIC2*) can hinder the binding of *GATA1* to the *BCL11A* enhancer by direct steric hindrance, thereby decreasing the interaction between the *BCL11A* promoter and its enhancer [56]. The RNA-binding protein LIN28B can suppress the *BCL11A* mRNA translation through direct interaction with ribosomes and *BCL11A* mRNA, suggesting that post-transcriptional regulation is involved in *BCL11A* regulation [45].

### LRF: another major*HBG* suppressor

The leukemia/lymphoma-related factor (*LRF*, encoded by *ZBTB7A*) belongs to the ZBTB family. Four ZFs exist in the *LRF*, and the pyrimidine-rich motif 5’ – CCCCTTCCCC-3’ which spans nucleotides − 203 to −194 in the fetal globin gene promoter was reported as the *LRF* binding element [31]. The *LRF* binds to the target DNA motif via its C-terminal C2H2 ZFs domain and can recruit a transcriptional repressive complex via its N-terminal BTB domain [57]. In 2016, the *LRF* was identified as another major suppressor of *HBG*, independent of the known key fetal globin repressor *BCL11A [*30]. Similar to *BCL11A*, the *LRF* also directly occupies the promoter of the fetal globin gene and then confers its inhibitory activity in concert with the NuRD repressor complex [30]. Since 2020, increasing evidence has highlighted the therapeutic potential of targeting *LRF (ZBTB7A)*-associated regulatory elements in hemoglobinopathies. Early studies identified the LRF-binding site as a promising genome-editing target for sickle cell disease [58]. More recent work has demonstrated that ZBTB7A/LRF and *BCL11A* binding sites contribute comparably to γ-globin reactivation, suggesting that CRISPR/Cas9-mediated disruption of either site may represent an effective strategy for treating β⁰-thalassemia/HbE disease [59]. Furthermore, emerging data support *ZBTB7A* as a compelling therapeutic target, where partial inhibition may achieve clinical benefit while minimizing adverse effects on erythroid differentiation [60]. Therefore, although simultaneous disruption of *LRF* and *BCL11A* may enhance HbF induction, its therapeutic application should be approached cautiously because of potential off-target effects and safety concerns. In contrast, targeted disruption of the *LRF* binding site alone, or in combination with the *BCL11A* binding site at the *HBG* promoters, may represent a more promising and clinically relevant strategy for future gene therapy development, although possible effects on erythroid differentiation should also be carefully evaluated.

## KLF1/EKLF: γ or β-globin gene activator

In 1995, A. C. Perkins et al. reported that knocking out the *KLF1/EKLF* in mice would lead to embryo death from lethal β-thalassemia^33^]. Later, A. C. Perkins et al. found that the silence of *HBG* expression was impaired if the *KLF1/EKLF* was absent [34]. Several reports indicated that haploinsufficiency for *KLF1/EKLF* can lead to HPFH [35, 38]. *KLF1/EKLF* mutations are found to be relatively more common in a thalassemia endemic region and can ameliorate the clinical and hematologic features of β-thalassemia [37]. In 2014, Shefali Soni et al. demonstrated that EKLF can interact with the histone cell cycle regulation defective homolog A (HIRA) and enable its selective recruitment of HIRA to the *HBB* promoter, thereby promoting the β-globin gene expression [61]. All these data suggest *KLF1/EKLF* as an adult β-globin gene activator protein.

Furthermore, *KLF1/EKLF*, which may be a regulatory target of c-Myb/*MYB* [62], is a dual regulator of the hemoglobin switching. *KLF1/EKLF* binds to the CACCC box in the promoter of *HBB* and then directly promotes *HBB* expression. *KLF1* can also bind to the promoter of *BCL11A* and stimulate *BCL11A* expression, resulting in the repression of the γ-globin gene [36]. Note that the CACCC-nucleotide sequence is shared by the ubiquitously expressed Sp1 and *KLF1*, which suggests that Sp1 may participate in the regulation of the hemoglobin switching [36]. In 2017, however, Beeke Wienert et al. reported a British HPFH mutation (−198T > C) that creates a *de novo KLF1/EKLF* binding site in the fetal γ-globin promoter, which is accompanied by reactivation of HBG expression. This observation indicates that forced recruitment of *KLF1/EKLF* to the HBG promoter via an engineered binding site can drive HBG transcription [32].

We can reasonably assume that the *KLF1/EKLF* is a general transcriptional activator in red cells. A study also supports this hypothesis. EKLF-GATA1 fusion protein, via GATA1-mediated DNA binding, can up-regulate δ and γ-globin gene expression in erythroid cells [63]. Nevertheless, the role of *KLF1* in the hemoglobin switching is complex and requires more in-depth and comprehensive study.

## GATA1: suppressor or activator

*GATA1* was originally thought to directly inhibit fetal globin gene expression and additionally contribute to *BCL11A*-mediated suppression [64]. *GATA1* can bind to the enhancer of *BCL11A* and regulate the expression of *BCL11A [*65]. Moreover, GATA1 can interact with BCL11A to form an inhibitory complex, thereby inhibiting *HBG* expression [28]. However, a study found that a natural mutation (−113 A > G) in HPFH creates a *de novo* recognition site for *GATA1* at the *HBG* proximal promoter, promoting *HBG* expression without affecting *BCL11A* binding [66]. More recently, disruption of a *BCL11A* suppressor binding motif in some forms of HPFH were reported to reactivate *HBG* by enabling the recruitment of the transcriptional activator NF-Y as well as *GATA1* to their own binding elements. In these forms of HPFH, the recruitment of *GATA1* becomes essential to activate γ-globin expression, while the *de novo* binding motif of *KLF1* or NF-Y is dispensable [67], suggesting *GATA1* as a crucial activator for *HBG*. Therefore, this conflicting phenomenon requires further investigation to gain a clear understanding of the exact role of *GATA1* in the hemoglobin switching.

## Other recently identified transcription factors/modulators to regulate the hemoglobin switching

In addition to the original HPFH-based studies, a strategy based on CRISPR or base editor screening has recently emerged to examine the regulation of the hemoglobin switching. These screening strategies are roughly categorized into two groups. The first group involves screening for novel TFs that regulate *HBG*, such as *ZNF410* and *NFI*. According to the regulation mechanism, these TFs can be further subdivided into two categories: factors that affect *HBG* expression by regulating or interacting with *BCL11A* or factors that regulate of *HBG* expression independent of *BCL11A*. The latter provides a new route to jointly target *BCL11A* and the newly discovered TFs to improve therapeutic efficiency. The second group involves screening for potential erythroid druggable targets or protein kinases that can regulate *HBG*, and which can be easily targeted by small molecule agents, such as *HRI*, *ETS2*, *HIF1α*, *VHL*, and *PTEN*. This screening strategy has extremely important value, because β-thalassemia is mainly prevalent in underdeveloped countries, and small molecule drugs have higher accessibility and practical significance compared with gene therapy. *ZNF410* is the only modulator discovered so far that represses fetal globin by only controlling a single gene, *CHD4*, encoding the NuRD nucleosome remodeler [68], suggesting that, unlike other suppressors, disruption of *ZNF410* to treat β-thalassemia can greatly avoid the toxic side effects resulting from interfering with a wide range of irrelevant targets of regulatory factors. *NFI* factors (*NFIA* and *NFIX*) were found to be a novel HbF inhibitor, partly because of their ability to increase *BCL11A* expression and partly by directly suppressing the *HBG1/2* genes [69]. The heme-regulated inhibitor *HRI*, an erythroid-specific kinase that controls protein translation, can increase *BCL11A* expression and thus promote γ-globin silence by activating *ATF4* [70, 71]. *ETS2*, encoded by *ERF*, inhibits *HBG* expression by (a) directly binding to DEBS and UEBS, which are specifically located downstream of *HBG1* and upstream of *HBG2*, respectively, and (b) epigenetic regulation, which is not reliant on previously defined erythroid transcriptional suppressors or complexes. The demethylation of the *HBG* promoter and attenuation of the interplay of the *HBG* promoter with *ERF* will occur when the *ERF* promoter is under the state of hypermethylation, followed by the result of the reactivation of *HBG* in β-thalassemia. Given its lack of effect on erythroid maturation and site-specific methylation with high efficiency, the *ERF* promoter-specific methylation through the dCas9-MQ1-sgRNA system represents a promising therapeutic target for β-hemoglobinopathies [72]. *PTEN* suppresses HbF through the negative control of AKT signaling, and the *PTEN* inhibitor (such as bpV) can significantly induce HbF [73]. Hypoxia-inducible factor 1α (*HIF1α*) is an activator to induce the γ- globin gene transcription via binding cognate DNA motifs in *BGLT3*, which is located 2.7 kb downstream of *HBG*, followed by chromatin opening, and recruiting transcriptional activators, thereby increasing the interplay between the fetal globin gene and LCR [74].

Some TFs, such as *FOG1* and *SOX6* [44, 75], interact with *BCL11A* to form a regulatory repressor complex; in cooperation with the NuRD complex, the epigenetic modulator of the globin gene, these factors may be involved in the regulation of the hemoglobin switching[28, 62, 76–78] (Table 2). Therefore, the role of these corepressors in the hemoglobin switching is also worthy of further research. *TR2* (*NR2C1*) and *TR4* (*NR2C2*), the orphan nuclear receptors, can recruit the direct repeat erythroid-definitive (DRED) repressor to the promoters of ε-globin and γ-globin [77]. Nuclear receptor corepressor-1 (*NCoR1*) can provide a scaffold for integrating the DNA binding and epigenetic enzyme components. Moreover, deubiquitinase *BAP1*, as a DRED repressor subunit, mediates the site-specific recruitment of *NCoR1* within the β-globin locus and thus plays an important role in *HBG* repression [79]. *ETO2* is a crucial regulator in erythropoiesis and globin gene switching through its repressive role in the *LDB1* complex, interacting with the NuRD complex, affecting the transcription factor and epigenetic environment, and ultimately restructuring chromatin organization [80]. Non-POU domain-containing octamer-binding protein (*NonO*) interacts directly with *SOX6* and bind directly to the ATGCAAAT motif in the fetal globin proximal promoter, thus acting as a novel suppressor of *HBG [*81].

The detailed regulatory mechanism of epigenetic modification in HbF silencing also helps to discover new therapeutic targets for β-thalassemia. Recently, the link of β-thalassemia phenotypes with *DNMT1* variants has been analyzed. The expression of γ-globin gene can be epigenetically depressed by a natural S878F mutation in the *DNMT1* BAH1 domain, thereby alleviating β-thalassemia severity[76]. Another epigenetic modulator, H3K4Me demethylase *KDM1A*/*LSD1*, suppresses γ-globin expression and promotes erythroid differentiation. The simultaneous inactivation of *LSD1* and *RUNX1* in erythroid progenitors can induce γ-globin synthesis without blocking erythroid differentiation [82]. Recently, modifying only a single CpG dinucleotide was proven to directly influence the binding of trans-acting factors and expression of a target gene in vivo. When the CpG dinucleotide of CGATA motif is unmethylated, the CGATA motif will become a binding motif of *GATA1*, an erythroid-specific factor whose typical binding sites are T/AGATA [83]. As the epigenetic regulatory enzymes are generally druggable, the study of epigenetics in HbF is of great value, a feature which will help the largest number of thalassemia patients in developing countries to obtain cheap but effective treatment.

### Hypotheses model of the hemoglobin switching

Two basic hypotheses attempt to explain the mechanism of the hemoglobin switching: autonomous silencing and gene competition.

The autonomous silencing hypothesis postulates that the globin gene would be silenced automatically as the development proceeds, and all the motifs responsible for silencing gene expression exist within the canonical gene or the nearby sequences [84]. The CACCC box was the major proximal fetal gene promoter element that participates in *HBG* silencing [85]. This mechanism mainly accounts for the silencing of the ε-gene and is the principal mechanism that turns off fetal globin gene expression. But gene competition also contributes to silence the fetal globin gene expression [20].

**Fig. 3:**
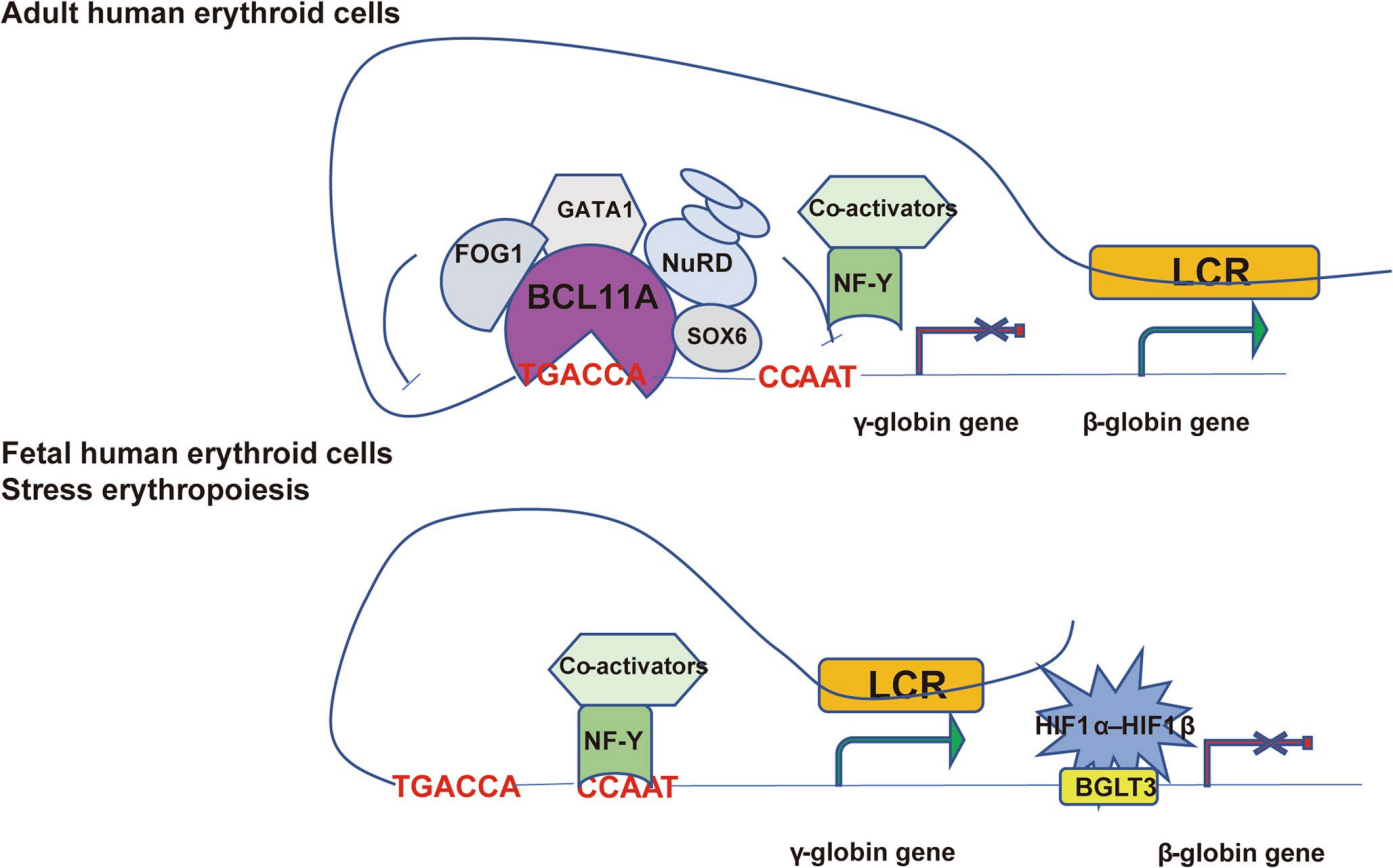
The competitive mechanism hypothesis for the hemoglobin switching

Gene competition assumes that the β- and γ-globin genes compete for interplay with the LCR [86] (Fig. 3). At a given time, the LCR interplays with only one promoter of globin genes and “flip-flops” between different globin promoters, relying on the stage of development [87]. During the fetal stage, the interplay between the γ-globin gene and LCR is so preferential that the β-globin gene is silenced competitively. As development advances, the situation overturns. The β-gene interacts with the LCR dominantly and lasts for a lifetime. Some important TFs, such as *GATA1*, *BCL11A*, *LRF/ZBTB7A*, and *EKLF*/*KLF1*, are involved in this process, providing opportunities for therapeutic targeting in the treatment of hemoglobinopathies.

During the fetal stage, low or absent expression of BCL11A allows the γ-globin activator NF-Y, together with co-activators, to bind the γ-globin promoter and facilitate its interaction with the LCR, thereby promoting γ-globin expression. After birth, BCL11A is highly expressed in adult erythroid cells. In cooperation with co-repressors, BCL11A inhibits NF-Y binding through steric hindrance and favors interaction between the β-globin promoter and the LCR. Consequently, β-globin expression is activated, while γ-globin expression is competitively silenced. Under stress erythropoiesis, HIF1α reactivates γ-globin expression by binding to the BGLT3 region, thereby promoting interaction between the γ-globin promoter and the LCR.

## Genetic Abnormalities in β-thalassemia

In contrast to the deletion of large fragments or even the entire gene in α-thalassemia, β-thalassemia usually results from mutations that leads to single nucleotide substitution or the insertion or deletion of small fragments within the β-globin genes or their adjacent sequences. These mutations will cause reduced synthesis of the β-globin chain and imbalance between α-globin and β-globin chains. Thus far, over 350 β-thalassemia mutations have been reported in HbVAR database [88].

The key pathophysiology of thalassemia entails the imbalance between α-like and β-like globin chain production [89], and the degree of the inequality between these two chains is closely tied to the severity of anemia. The excess free α-globin chains are highly unstable and will form aggerate and precipitate in red cells because of the deficient production of the β-globin chain [90], an occurrence which will further induces reactive oxygen species that damage the viability and maturation of erythroid precursors, leading to the premature hemolysis of circulating red cells and ineffective erythropoiesis [1, 91, 92]. Given the ubiquitin–proteasome system mediated by the α-hemoglobin–stabilizing protein (AHSP), the erythroid precursors in β-thalassemia can tolerate and detoxify a modest pool of free α-globin chain [93]. Once this system is impaired, the disease severity will increase.

Inducing increased production of the fetal globin chain after birth can substitute the β-globin chain with the γ-globin chain and thus alleviate the degree of inequality between the α-globin and β-globin chains, a notion which represents an inspiring and promising field to treat TDT.

## Strategy of Gene Therapy for β-thalassemia

Gene therapy, roughly divided into gene addition and gene editing, was quickly developed as a promising therapeutic tool for TDT in recent years. Gene addition, mainly referring to *HBB* restoration, is a process involving the separation and extraction of the patient’s own hematopoietic stem and progenitor cells (HSPCs), the introduction of the normal β-globin gene, and the subsequent autologous HSCT of these rectified HSPCs. At present, many ongoing clinical trials of gene therapy for β-thalassemia are based on this strategy (Table 3). By using autologous hematopoietic stem cells (HSCs), gene therapy technologically overcomes the difficulty of HLA matching and is correlated with a lower risk of graft rejection and graft-versus-host disease and infection compared with allo-HSCT [89]. A lentiviral vector including normal functional *HBB* and key regulatory elements is needed to transduce HSCs in gene addition approaches. The LentiGlobin BB305 vector has been demonstrated in Phase 1 and 2 studies to led to the discontinuation of blood transfusions for 68% of patients with TDT and for the remaining part who continued transfusions, annualized transfusion requirements were decreased by 73% [94]. Another clinical trial using the GLOBE lentiviral vector and intraosseous infusion of modified HSCs showed promising but limited data [95]. Recently, a new *HBB* restoration strategy was proposed by M. Kyle Cromer et al. They leveraged a Cas9/AAV6-mediated gene editing method that can displace the entire Hemoglobin Subunit Alpha 1 (*HBA1*) gene with a normal full-length β-globin gene in patient-derived HSPCs, an approach which reintroduces normal adult globin tetramers sufficiently and also normalizes β-globin: α-globin mRNA, and protein ratios [96]. Given the random integration of lentiviruses across the genome in these gene addition approaches, the potential risk of insertional mutagenesis via activation of an oncogene and inactivation of a tumor suppressor gene should be noted.

**Table 3:** Gene therapy and genome editing clinical trials for β-thalassemia

Strategy	Modality	Sponsor	NCT Number Phase	Number Enrolled	Status
β-Like globin gene replacement	Lentiviral vector self-inactivating lentiviral vector to correct the defective gene	Shenzhen Geno-Immune Medical Institute	NCT03351829 Not Applicable	20	Unknown †
	GLOBE encoding the human beta globin gene (OTL-300)	1)IRCCS San Raffaele 2)Telethon Institute for Gene Therapy (OSR-TIGET)	NCT03275051 Not Applicable	9	Active, not recruiting
	GLOBE lentiviral vector encoding for the human beta-globin gene	1)IRCCS San Raffaele 2)Fondazione Telethon 3)Orchard Therapeutics	NCT02453477 Phase 1 Phase 2	10	Unknown †
	lentiviral vector encoding for beta-globin gene.	1)Nanfang Hospital of Southern Medical University 2)Guangdong Yike Gene Science and Technology CO.,Ltd	NCT03276455 Phase 1 Phase 2	10	Unknown †
	LentiGlobin BB305 lentiviral vector encoding the human βA-T87Q-globin gene	bluebird bio	NCT01745120 Phase 1 Phase 2	19	Completed Has results*
	LentiGlobin BB305 Drug Product (autologous CD34 + cell-enriched population that contains cells transduced with LentiGlobin BB305 lentiviral vector encoding human βA-T87Q-globin)	bluebird bio	NCT03207009 Phase 3	18	Active, not recruiting
	LentiGlobin BB305 Drug Product (autologous CD34 + cell-enriched population that contains cells transduced with LentiGlobin BB305 lentiviral vector encoding human βA-T87Q-globin)	bluebird bio	NCT02906202 Phase 3	23	Completed
	Following myeloablative conditioning with IV busulfan for 4 consecutive days (dose may be adjusted as per protocol) and subsequent daily monitoring of busulfan levels for confirmation of adequate washout, a single dose cluster of differentiation (CD) 34 + cells/kg LentiGlobin BB305 Drug Product was administered to participants by IV infusion.	bluebird bio	NCT02151526 Phase 1 Phase 2	7	Completed Has results*
	Genetic: BD211 Drug Product Transplantation of Autologous CD34 + Stem Cells Transduced to BD211 finished Product with a Lentiviral Vector coding βA-T87Q-Globin.	1)Shanghai BDgene Co., Ltd. 2)920th Hospital of Joint Logistics Support Force of People’s Liberation Army of China	NCT05015920 Not Applicable	10	Recruiting
	Autologous CD34 + cells transduced with TNS9.3.55 encoding the human ß-globin gene.	Memorial Sloan Kettering Cancer Center	NCT01639690 Phase 1	10	Active, not recruiting
	lentiviral vector LentiHBBT87Q encoding the human β-globin gene.	1)BGI-research 2)Shenzhen Children’s Hospital	NCT04592458 Phase 1	10	Not yet recruiting
HbF Induction	Nuclease/target				
	ST-400 Investigational product is composed of autologous CD34 + hematopoietic stem/progenitor cells that are genetically modified ex vivo at the erythroid-specific enhancer of the BCL11A gene	1)Sangamo Therapeutics 2)Sanofi 3_Sangamo Therapeutics	NCT03432364 Phase 1 Phase 2	5	Completed
	EDIT-301, consists of patient-derived CD34 + HSPCs edited at the gamma globin gene (HBG1 and HBG2) promoters, by a highly specific and efficient proprietary engineered AsCas12a nuclease	Editas Medicine, Inc.	NCT05444894 Phase 1 Phase 2	6	Recruiting
	CRISPR-Cas9-mediated gene editing of the BCL11A enhancer	1)Bioray Laboratories 2)Xiangya Hospital of Central South University 3)PLA 923 Hospital	NCT04211480 Not Applicable	6	Active, not recruiting
	γ-globin reactivated autologous HSCs will be manufactured using Glycosylase Base Editors(BRL-103)	1)Bioray Laboratories 2)First Affiliated Hospital of Guangxi Medical University	NCT05442346 Not Applicable	5	Not yet recruiting
	CTX001 (autologous CD34 + hHSPCs modified with CRISPR-Cas9 at the erythroid lineage-specific enhancer of the BCL11A gene)	1)Vertex Pharmaceuticals Incorporated 2)CRISPR Therapeutics	NCT03655678 Phase 2 Phase 3	45	Active, not recruiting
Others**	gene correction of HBB in patient-specific iHSCs using CRISPR/Cas9	Allife Medical Science and Technology Co., Ltd.	NCT03728322 Early Phase 1	12	Unknown †

† The study has passed its completion date, and its status has not been verified in more than two years

*The detailed results can be found in the Clinicaltrials.gov

**The strategy cannot be categorized because of the vague description in Clinicaltrials.gov

**Fig. 4:**
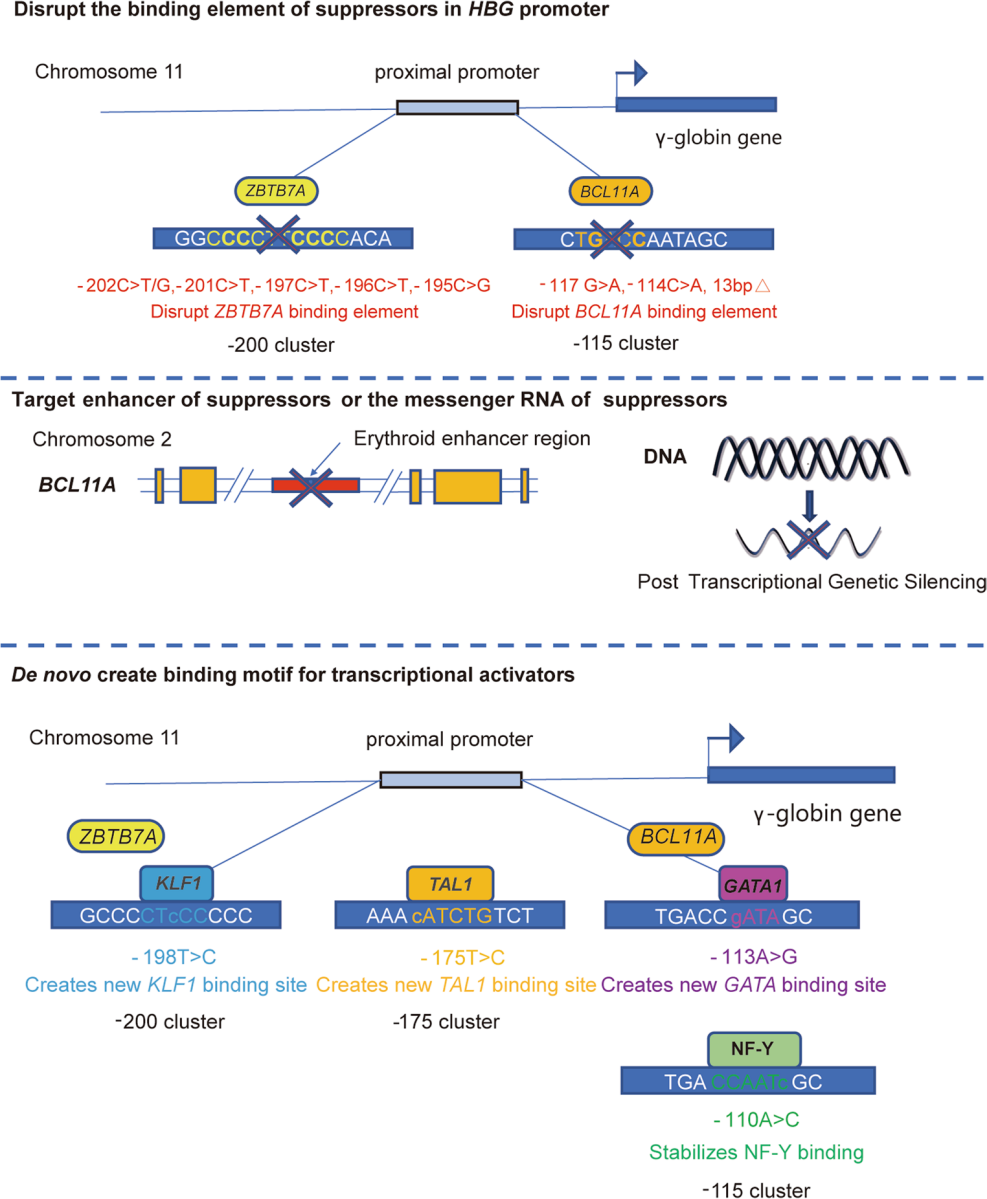
Targets of gene therapy for β-thalassemia

To reactivate fetal globin expression, gene editing targets can be the promotor of *HBG*, disrupting the binding element of suppressors, the enhancer or the messenger RNA of suppressors, such as *BCL11A [*28] and *LRF [*30]. Furthermore, *de novo* creating binding motif for transcriptional activators [67]or together with overexpressing these activators in transfection vectors, such as *GATA1* [66], NF-Y [97], *KLF1* [32], *TAL1* [98] is also a worthy strategy (Fig. 4). Disruption of the binding sequence of the suppressor or simultaneously creating the binding sequence of the activator *de novo* in the *HBG* promotor without perturbing other target motifs of *HBG* suppressors, such as *BCL11A*, is relatively safer than directly targeting the expression of suppressors, because the former preserves the other essential unknown functions of these transcriptional suppressors. Thus far, *BCL11A*, the main repressor of the human γ-globin gene, has been the major target in gene editing strategies, but *LRF*, alone or together with *BCL11A*, is also a disrupting target worth exploring in the future [31].

The top of the panel shows the strategy that disrupts the binding element of key suppressors of *HBG*, such as *BCL11A* and *LRF*. The red words represent the natural mutations that can block the binding of these suppressors to their target motif in HPFH. The middle of the panel represents a strategy that disrupts the erythroid-specific enhancer of *BCL11A* (Left) or represses *BCL11A* expression via post-transcriptional genetic silencing (Right). The bottom of the panel illustrates a new strategy for reactivating the *HBG* by *de novo* creating a binding motif or stabilizing the binding to the target motif for transcriptional activators, such as *KLF1*, *TAL1*, *GATA1* and NF-Y. The words below the boxes represent the natural mutations in HPFH.These strategies can be combined flexibly to optimize the efficacy and safety of gene therapy.

Gene therapy has shown exciting and surprising effects in treating β-thalassemia, but considerable effort is still needed to perfect gene therapy protocols to improve safety, efficiency, and simplicity. CRISPR/Cas9 technology is an earlier gene editing strategy for the treatment of β-thalassemia, but it carries a potential carcinogenic risk because it causes DNA double-strand breaks (DSB), whose error-prone repair may result in genomic instability and oncogenic alterations [99, 100. To overcome these unfavorable factors, David Liu’s laboratory fused the catalytically inactive Cas9 with deaminase to establish an efficient and precise single-base editing systems, namely, cytosine base editors (CBEs) and adenine base editors (ABEs), which can realize site-directed single base transformation without causing DSB [101, 102]. ABE has been used to correct a severe and common β-thalassemia mutation, IVS1-110 (G > A), achieving approximately 80% gene correction in HSPCs, with restoration of β-globin expression and improved erythroid differentiation in the absence of DNA double-strand breaks [103]. Furthermore, base editing technology was employed to dissect a *HBG* cis-acting element, demonstrating that base editing of the fetal globin gene promoter represents a universal, safe, and promising strategy for the treatment of β-hemoglobinopathies [104]. Yuxuan Wu’s group introduced nucleotide substitutions in the *HBG* promoter or *BCL11A* enhancer in β-thalassemia patient-derived CD34^+^ HSPCs to successfully upregulate fetal globin expression; moreover, they directly rectified IVS II-654 mutations in the HSPCs from thalassemia patients and Hemoglobin E (HbE) by developing ABE8e-SpRY, a near-PAMless ABE variant, suggesting the promising potential of ABE-mediated base editing in the treatment of hereditary monogenic hematological diseases [105]. However, we must also be aware that β-thalassemia generally has multiple site mutations at the same time in *HBB*, but the current base editors can only realize the four transitions (G→A, C→T, T→C, and A→G) and cannot achieve other types of base mutations and base insertion-deletion mutations. This limitation restricts the application of such base editors in the treatment of β-thalassemia. In 2019, David Liu’s group developed a new versatile precise gene editing tool Prime Editors (PEs). PE can effectively achieve the free conversion of all 12 single bases and realize the precise insertion and deletion of multiple bases (up to 44 bp can be inserted, and 80 bp can be deleted) without needing donor DNA or DSB, a feature which brings about revolutionary changes in the field of gene editing [106]. Based on the CRISPR/Cas9 system, PE is modified in two aspects: the first aspect involves modifying a single-stranded guide RNA (sgRNA), which has an RNA sequence added to its 3’ end to generate the newly obtained RNA called pegRNA. The second aspect entails fusing Cas9 nickase (H840A mutant, which only cuts the target DNA strand containing PAM) with reverse transcriptase to obtain a new fusion protein. PE is a breakthrough in the field of precise gene editing given its great potential in the free conversion of single bases and the addition and deletion of small fragments of multiple bases, which will greatly promote biomedicine and clinical gene therapy research. Compared with previous gene editing systems, the prime editing gene editing system avoids the occurrence of DSB, improves gene editing efficiency, and expands the scope of application. However, the large size of the components of prime editing limits its clinical application in vivo. Furthermore, overexpression of the main component, reverse transcriptase, may pose safety concerns related to potential cellular toxicity and off-target effects, warranting further investigation [106]. A vectorized prime editing system has also been developed recently to directly correct the SCD mutation in HSCs in vivo in a SCD mouse model (CD46/Townes mice) [107]. More recently, emerging base-editing strategies have demonstrated the ability to precisely correct β⁰-thalassemia mutations in hematopoietic stem cells, with high editing efficiency, functional recovery of β-globin, and promising preclinical safety [108].

Strategy optimization can be conducted at every step of the process, such as during the design of the vector, Cas9 protein, and guide RNA (gRNA) and off-target assessment. In addition to the technical updates above, several other gene therapy strategies to enhance efficacy have also recently been proposed. The *KLF1*-*GATA1* fusion protein was designed to interplay with the δ-globin gene (*HBD*) promoter and induce *HBG* expression in human primary CD34^+^ cells [109]. CRISPR-Cas genome editing can be enhanced by chemically modified gRNAs in human primary cells [110]. *HBG* reactivation through joint introduction of cis and trans mutations shows higher HbF expression than single target editing in vitro and in vivo, bringing great clinical benefit to patients with severe β-thalassemia phenotype [111]. Improving the safety of gene editing and reducing the off-target effects are also important aspects of gene therapy. Recently, CRISPRme, a variant-aware off-target assessment tool which considers indel genetic variants and single-nucleotide polymorphism was developed to nominate and prioritize off-target sites, demonstrating promise in clinical trials for β-thalassemia and SCD and providing a powerful approach for comprehensive off-target nomination [112].

### Challenges and Future Outlook

Recently, with the deepening understanding of the hemoglobin switching mechanism, gene therapy targeting hemoglobin switching regulators have achieved stunning results, becoming one of the promising methods to cure thalassemia. However, we should acknowledge that gene therapy faces numerous problems and challenges.

First, long-term observation is still needed to comprehensively evaluate the safety of gene therapy, especially its cancer risk. Second, the high cost and technical complexity of gene therapy limit its widespread use. Inequality in access to medical resources around the world remains a major challenge and concern for β-thalassemia treatment, as most β-thalassemia patients live in underdeveloped areas. That is, despite the advent of new and effective treatments, they may be unavailable to these people in need. Thus, affordability is advised to be a key and even the first consideration for current and future therapeutic approaches, such as for screening druggable targets. Then, these treatments can be easily incorporated into the local therapeutic systems and be maintained by those resource-limited countries. Furthermore, establishing a special organization under the unified deployment management of the gene therapy community to guarantee national and international cooperation, stable financial support, and data sharing may help benefit most β-thalassemia patients. More importantly, the popularization of pre-diagnosis and genetic counseling in developing countries will fundamentally reduce TDT from the root.

## Data Availability

No datasets were generated or analysed during the current study.

## Abbreviations

AHSP: α-hemoglobin–stabilizing protein
ABEs: Adenine base editors
BCL11A: B-cell lymphoma/leukemia 11 A
bp: Base pairs
CBEs: Cytosine base editors
DSB: DNA double-strand breaks
DNMT3A: DNA methyltransferase 3 A
DRED: Direct repeat erythroid-definitive
EHMT: Euchromatic histone lysine methyltransferase
GWAS: Genome-wide association studies
gRNA: Guide RNA
HBA1: Hemoglobin subunit alpha 1
HBB: β-globin gene
HBD: δ-globin gene
HbE: Hemoglobin E
HbF: Fetal hemoglobin
HBG: γ-globin gene
HIRA: Histone cell cycle regulation defective homolog A
HSCT: Hematopoietic stem cell transplantation
HSPCs: Hematopoietic stem and progenitor cells
HSCs: Hematopoietic stem cells
HSs: Hypersensitive sites
HPFH: Hereditary persistence of fetal hemoglobin
KLF1/EKLF: Krüppel-like factor 1
KDM1A: Lysine demethylase 1 A
LSD1/CoREST: Lysine-specific demethylase 1 and repressor element-1 silencing transcription factor corepressor 1
LCR: Locus control region
LRF: Leukemia/lymphoma-related factor
NTDT: Non–transfusion-dependent β-thalassemia
NuRD: Nucleosome remodeling and deacetylase
NCoR1: Nuclear receptor corepressor-1
NonO: Non-POU domain-containing octamer-binding protein
PEs: Prime Editors
SCD: Sickle cell disease
sgRNA: Single-guide RNA
TDT: Transfusion-dependent β-thalassemia
TFs: Transcription factors
ZBTB7A: Zinc Finger and BTB Domain Containing 7 A
ZFs: Zinc fingers
